# Starvation-induced activation of ATM/Chk2/p53 signaling sensitizes cancer cells to cisplatin

**DOI:** 10.1186/1471-2407-12-571

**Published:** 2012-12-04

**Authors:** Yandong Shi, Emanuela Felley-Bosco, Thomas M Marti, Katrin Orlowski, Martin Pruschy, Rolf A Stahel

**Affiliations:** 1 Laboratory of Molecular Oncology, Zürich, Switzerland; 2 Department of Radiation Oncology, University Hospital Zürich, University of Zürich, Zürich, Switzerland

**Keywords:** Serum starvation, short-term food starvation (STS), cisplatin therapy, ATM/Chk2/p53 signaling, AMPK

## Abstract

**Background:**

Optimizing the safety and efficacy of standard chemotherapeutic agents such as cisplatin (CDDP) is of clinical relevance. Serum starvation in vitro and short-term food starvation in vivo both stress cells by the sudden depletion of paracrine growth stimulation.

**Methods:**

The effects of serum starvation on CDDP toxicity were investigated in normal and cancer cells by assessing proliferation, cell cycle distribution and activation of DNA-damage response and of AMPK, and were compared to effects observed in cells grown in serum-containing medium. The effects of short-term food starvation on CDDP chemotherapy were assessed in xenografts-bearing mice and were compared to effects on tumor growth and/or regression determined in mice with no diet alteration.

**Results:**

We observed that serum starvation in vitro sensitizes cancer cells to CDDP while protecting normal cells. In detail, in normal cells, serum starvation resulted in a complete arrest of cellular proliferation, i.e. depletion of BrdU-incorporation during S-phase and accumulation of the cells in the G0/G1-phase of the cell cycle. Further analysis revealed that proliferation arrest in normal cells is due to p53/p21 activation, which is AMPK-dependent and ATM-independent. In cancer cells, serum starvation also decreased the fraction of S-phase cells but to a minor extent. In contrast to normal cells, serum starvation-induced p53 activation in cancer cells is both AMPK- and ATM-dependent. Combination of CDDP with serum starvation in vitro increased the activation of ATM/Chk2/p53 signaling pathway compared to either treatment alone resulting in an enhanced sensitization of cancer cells to CDDP. Finally, short-term food starvation dramatically increased the sensitivity of human tumor xenografts to cisplatin as indicated not only by a significant growth delay, but also by the induction of complete remission in 60% of the animals bearing mesothelioma xenografts, and in 40% of the animals with lung carcinoma xenografts.

**Conclusion:**

In normal cells, serum starvation in vitro induces a cell cycle arrest and protects from CDDP induced toxicity. In contrast, proliferation of cancer cells is only moderately reduced by serum starvation whereas CDDP toxicity is enhanced. The combination of CDDP treatment with short term food starvation improved outcome in vivo. Therefore, starvation has the potential to enhance the therapeutic index of cisplatin-based therapy.

## Background

Cisplatin (CDDP) is a standard therapeutic agent for the treatment of various solid tumors. Its efficacy however may be limited by patients’ tolerance [1]. The aim of our investigation was to identify ways to increase the efficacy of CDDP for cancer killing while enhancing the tolerance of normal cells.

Tumor cells are exposed to numerous cellular stresses, such as oncogene-induced genotoxic stress [2], oxidative stress [3], and metabolic stress [4], which are not affecting normal cells. Thus, tumor cells are more dependent on stress support pathways for survival compared to normal cells. Therapy targeting the stress response pathways, which can principally be reached through inhibiting the activity of these pathways or through overloading stress to overwhelm these pathways, may be specifically detrimental to cancer cells while sparing normal cells [5]. For example it has been shown that interfering the cellular response to oxidative stress by a small molecule selectively kills cancer cells [6], and that targeting replicative stress response pathway resulted in specific killing of oncogene-driven tumors [7].

Serum starvation in vitro and short-term food starvation (STS) in vivo have been demonstrated to reduce the levels of growth factors [8-11]. In normal cells, depletion of paracrine growth factors reduces the activity of proliferation-stimulating signaling pathways and reduces basal cellular metabolism subsequently leading cells to enter a proliferation-quiescent status [12]. In contrast, starvation may specifically induce stress in cancer cells because cancer cells struggle to adapt to the loss of external growth factors by adjusting autonomous growth stimulation and reprogramming their metabolism thereby maintaining continuous proliferation [13].

We investigated whether starvation affected the outcome of CDDP therapy in vitro and in animal models. We show that serum starvation activates the cellular DNA damage response pathway specifically in cancer cells. Our data suggest that starvation has a potential to increase CDDP-induced toxicity in cancer cells and simultaneously enhance the tolerance of normal cells to CDDP treatment.

## Results

### Serum starvation sensitizes ZL55cancer cells to CDDP

**Figure 1:**
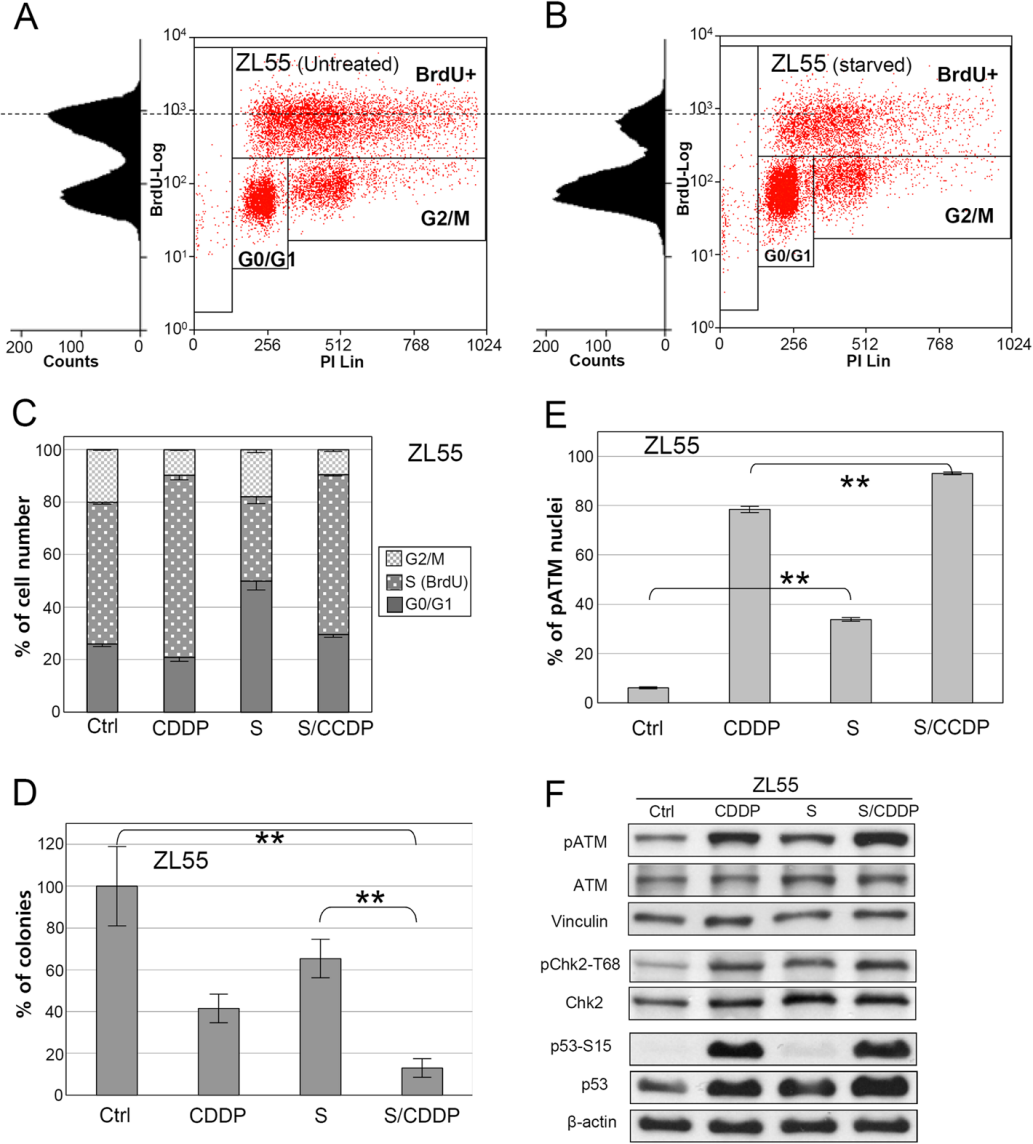
**Serum starvation sensitizes ZL55cancer cells to CDDP.** Results of flow cytometry analysis of ZL55 cells untreated (**A**) or serum-starved for 24 hours (**B**) are shown. **C**, the quantification of flow cytometry results of ZL55 cells of the untreated control, or treated with CDDP alone, serum starvation alone, or both together is shown (n=3). **D**, colony formation assay was performed after control treatment (Ctrl), treatment with CDDP or serum starvation (S) alone, and after the combined treatment (S/CDDP) (n=6, ** p<0.001). The quantification of the anti-pATM-S1981 immuno-staining of ZL55 cells (** for P<0.001) is shown in (**E**) (n=3). Results of Western blot with antibodies against pATM-S1981 (pATM), ATM, phospho Chk2-T68 (pChk2-T68), Chk2 and p53-Ser15 (p53-S15) and p53 for protein extracts are shown in (**F**).β-Actin and vinculin were used as loading controls.

FACS analysis of BrdU pulse-labeled ZL55 cancer cells revealed that the fraction of cells in S-phase were decreased by 40% after 24 hours serum starvation compared to untreated control (Figure 1A-C). BrdU incorporation in starved cancer cells was also reduced suggesting a reduction of DNA replication in the remaining S-phase cells (Figure 1B to 1A). When cancer cells were exposed to CDDP in serum-starved conditions, an increased sensitivity was observed: the combined treatment of CDDP and serum starvation additively reduced the clonogenicity of human mesothelioma ZL55 cells in comparison to either treatment alone (Figure 1D).

To explore the mechanism of the CDDP sensitization by serum starvation, the response of cancer cells to serum starvation was examined in more detail. ATM is the key component in cellular stress responses to DNA damage and oxidative stress [14,15]. In ZL55 cells serum starvation induced a five-fold increase of nuclei positive for Serine1981-phosphorylated ATM (Ataxia Telangiectasia Mutated), compared to untreated control (Figure 1E, and Additional file 1: Figure S1A and S1C). The starvation-induced activation of ATM in ZL55 cells was confirmed by Western blot analysis (Figure 1F), which revealed a 40% increase in phosphorylated ATM. Chk2 and p53 are downstream targets of ATM [14,15]. In addition to the activation of ATM, enhanced phosphorylation of Chk2 and 50% elevation of p53 protein levels were also observed in starved ZL55 cells. We noticed that the serine15-phosphorylation status of p53 in cancer cells is not affected by serum starvation (Figure 1F).

CDDP-triggered high levels of pATM-S1981 in ZL55 cells, which was further increased significantly (P<0.001)in the presence of serum starvation (Figure 1E and 1 F, and Additional file 1: Figure S1B and S1D)resulting in more than 90% pATM-S1981 positive nuclei under immunofluorescent microscopy.

In summary, serum starvation and CDDP both activate ATM/Chk2/p53 signaling in ZL55 cancer cells and when combined result in an enhanced activation of the signaling and sensitization of cancer cells to CDDP.

### Activation of ATM/p53 is required for the sensitization of cancer cells to CDDP

**Figure 2:**
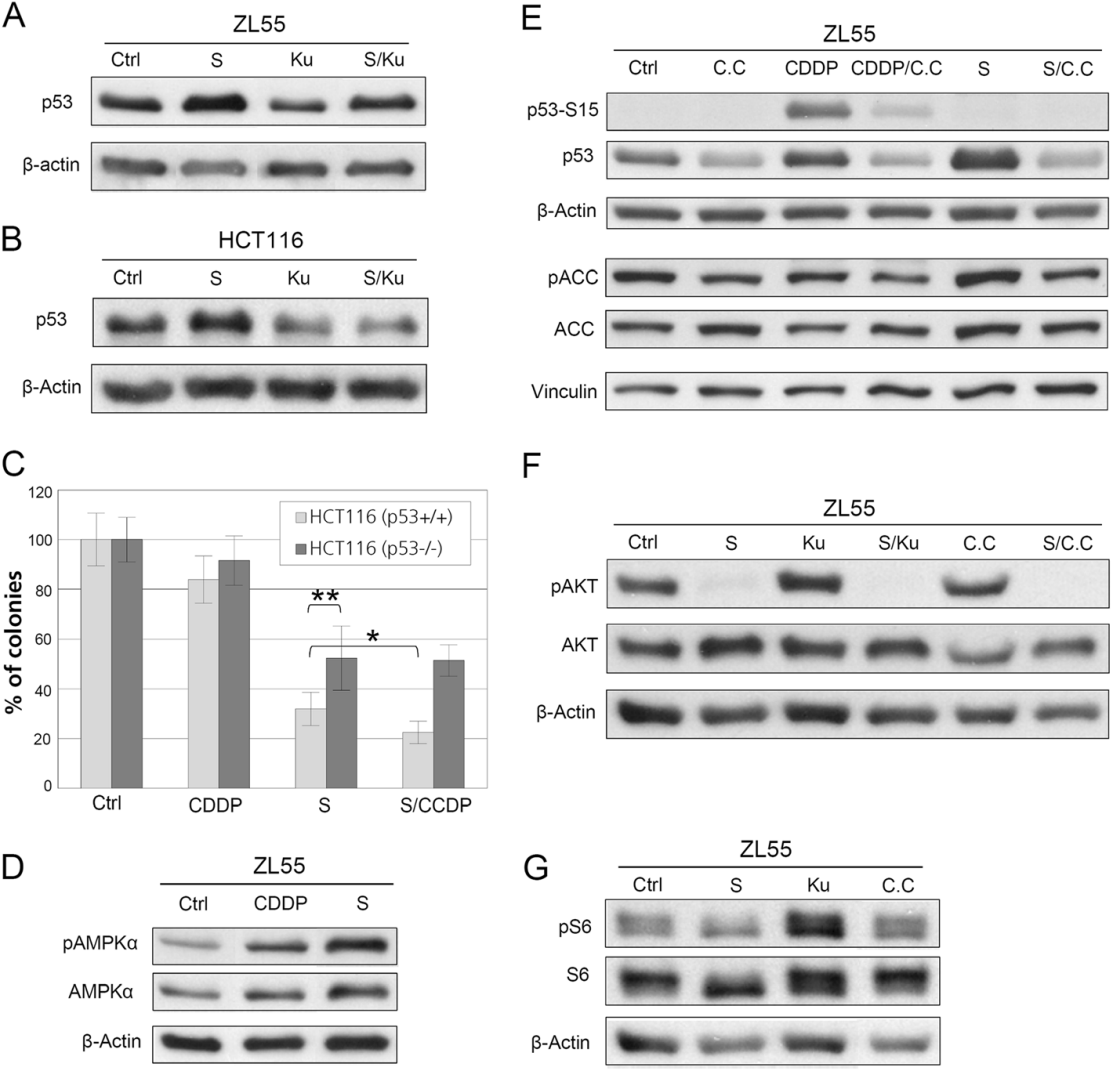
**Serum starvation-induced activation of ATM/p53 is required for the CDDP sensitization of cancer cells and both ATM and AMPK are necessary for the activation of p53.** Western blot results with antibodies against p53 in protein extracts from ZL55 cells (**A**) and HCT116 (**B**) treated by Ku-55933 (Ku) alone, serum starvation alone, or both together are shown. Results of colony formation assay for the untreated control HCT116-p53^−/−^ and HCT116-p53^+/+^ cells and those treated by CDDP alone, serum starvation alone (S), or both together p53-knockout (n=6, * P<0.02, ** P<0.01, significances were calculated with two-tailed *t*-Test) are shown in (**C**). Results of Western blot with antibodies against pAMPK and AMPK in protein extracts from untreated control ZL55 cells, or treated with CDDP alone or serum starvation alone are shown in (**D**). Results of Western blot with antibodies against p53-Ser15 (p53-S15), p53, pACC and ACC in protein extracts from the untreated control ZL55 cells and those after treatment with Compound C (C.C) alone, CDDP alone, CDDP and C.C together, serum starvation alone, or serum starvation and C.C together are shown in (**E**). Results of Western blot with antibodies against phospho-AKT-Ser473 (pAKT) and AKT in protein extracts from the untreated controlZL55 cells and after treatment with serum starvation alone, Ku55933 alone, serum starvation andKu55933 together, Compound C (C.C) alone, or serum starvation and C.C together are shown in (**F**). Results of Western blot with antibodies against phospho-S6-Ser235/236 (pS6) andS6 in protein extracts from the untreated control ZL55 cells and after treatment with serum starvation alone, Ku55933 alone or Compound C (C.C) alone are shown in (**G**). β-Actin and vinculin were used as loading controls.

The requirement for ATM/p53 activation in the serum starvation-mediated CDDP sensitization of cancer cells was examined. We observed that the serum starvation-induced activation of p53 is ATM-dependent because it was inhibited by Ku-55933, a specific inhibitor of ATM [16] (Figure 2A). Similar to the situation in ZL55 cells, serum starvation resulted in accumulation of p53 in an ATM-dependent manner in human colorectal cancer HCT116 cells (Figure 2B). A functional role for p53 in the serum starvation-mediated CDDP sensitization was investigated by comparing p53 deficient (p53−/−) versus p53 proficient (p53+/+) HCT116 cells [17] (functionally characterized for response to CDDP in Additional file 1: Figure S2). Serum starvation-induced reduction of clonogenicity was significantly suppressed in p53-deficient HCT116 cells in comparison to p53-proficient HCT116 cells. The combination of serum starvation and CDDP further decreased clonogenicity in the p53-proficient cells, but not in p53-deficient cells (Figure 2C). These data obtained with the colorectal cancer cells suggest that the serum starvation-mediated sensitization of cancer cells to CDDP is dependent on ATM/p53 activation.

### Serum starvation-triggered phosphorylation of AMPK is required for the stabilization of p53 in cancer cells

AMPK is an important regulator in cellular stress responses [18] and activates p53 in response to glucose starvation [19]. The phosphorylation on the Thr-172 of AMPKα (pAMPK-Thr172) is the crucial step of AMPK activation [18]. The involvement of AMPK in the cellular response to serum starvation was examined in cancer cells. Increased levels of pAMPK-Thr172 were observed after serum starvation (Figure 2D). In agreement with the activation of AMPK, elevated phosphorylation of acetyl-CoA carboxylase (ACC), one of the downstream targets of AMPK [20], was detected in starved ZL55 cells as well, which was suppressed in the presence of compound C, the specific inhibitor of AMPK [21] (Figure 2E). Compound C inhibited the activation of p53 induced by both CDDP and serum starvation (Figure 2E), indicating a general role of AMPK in the activation of p53 in cancer cells. Consistent with studies indicating that inhibition of IGF1 signaling is required for starvation-mediated sensitization of cancer cells to therapeutic drugs [22,23], we observed a decrease of AKT phosphorylation upon serum starvation (Figure 2F). The latter was not affected by ATM inhibitor Ku55933 or AMPK inhibitor Compound C, indicating that the inactivation of PI3K pathway and the activation of p53 by AMPK/ATM are likely independent mechanisms.

In addition, we verified the possible interference of ATM and AMPK inhibitors on mTOR signaling by testing their effects on the phosphorylation of S6, which is a target downstream of mTOR. While serum starvation strongly inhibited the phosphorylating of S6, ATM inhibitor Ku55933 and AMPK inhibitor Compound C did not show any effect on the S6 phosphorylation (Figure 2G), suggesting that ATM inhibitor (Ku55933) and AMPK inhibitor (Compound C) were quite target-specific and do not interfere activity of mTOR signaling.

### Short-term food starvation sensitizes human mesothelioma xenografts to CDDP

**Figure 3:**
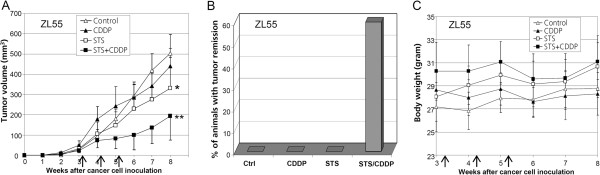
**Starvation sensitizes human mesothelioma xenografts to CDDP in vivo.**. **A**, growth curves of ZL55 tumors in the untreated control animal group and those treated with CDDP, STS, or both together (n=5/group) (* P<0.05; ** P<0.01). **B**, complete tumor remission is observed only with the combined treatment. **C**, curves of average body weight of animals with ZL55 tumors in different groups during and after the treatments. Arrows in (**A**) and (**C**) indicate the time points of individual treatments.

To investigate combined effect of starvation with CDDP in vivo*,* short-term food starvation (STS) was implemented [22-24]. ZL55 cells were subcutaneously injected into nude mice. Tumor-bearing animals were treated with the standard dose of CDDP (3 mg/kg) in the presence or absence of STS, or with STS alone once per week for three weeks. No significant inhibition of tumor growth was observed when CDDP was administrated alone. A mild (P<0.05) delay of tumor growth by STS alone was observed (Figure 3A). However, a dramatic (P<0.01) inhibition of tumor growth was observed when mice were treated with the combination of CDDP and STS. The average tumor volume was reduced by more than 60% three weeks after treatment, compared with untreated controls (Figure 3A). Tumors continued growing for 4 weeks after ending of the combination treatment. Then, tumors started to regress. During the 8th to 10th week after the treatment, complete remission was observed in 60% combination-treated animals. Animals with complete tumor remission were kept for additional at least 4 weeks, and no recurrence was observed. No remission was observed in any other groups at the end of a 16 weeks follow-up period (Figure 3B).

Animals lost 15% in average body weight during STS, but they regained most of the lost body weight during the next day after STS (Additional file 1: Figure S3).No apparent effect on the evolution of body weight was observed overlong term duration (Figure 3C). Thus, our data suggest that STS with water ad libitum is tolerable for animals and strongly sensitizes mesothelioma tumors to CDDP in vivo.

### STS sensitizes human lung carcinoma A549 xenografts to CDDP

**Figure 4:**
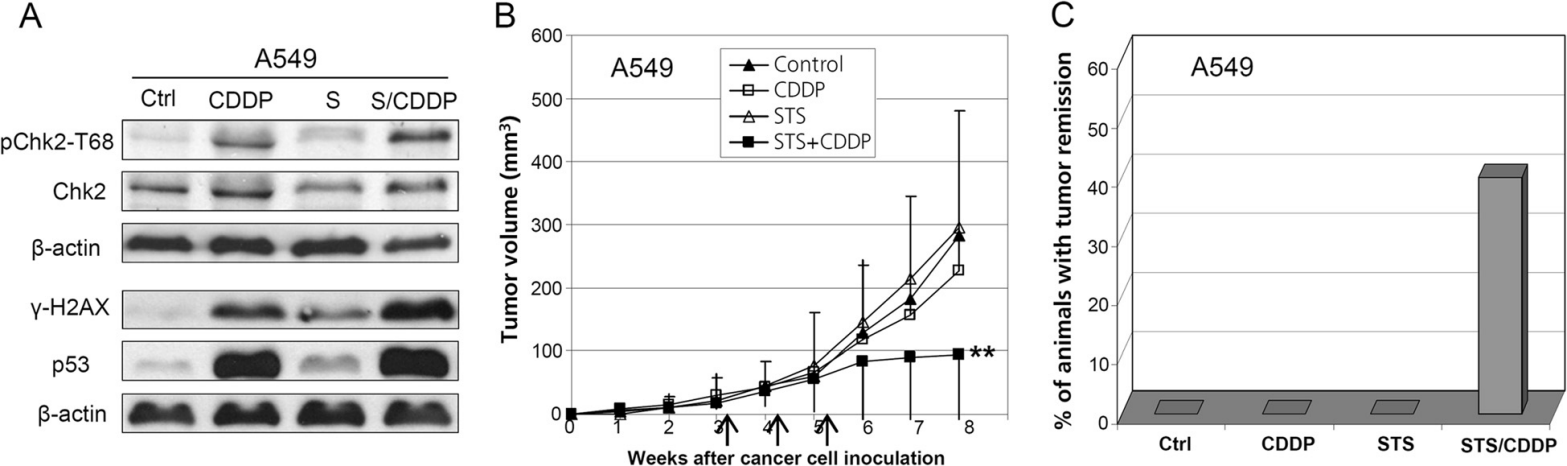
**Starvation sensitizes human lung carcinoma A549 xenografts to CDDP in vivo.A**, Western blot results with antibodies against phospho Chk2-T68, Chk2, γ-H2AX and p53 for protein extracts from untreated control A549 cells and those treated with CDDP alone, serum starvation alone, or both together are shown. β-Actin was used as loading control. **B**, growth curves of A549 tumors in the untreated control animal group and those treated with CDDP, STS, or both together (n=6/group) (** P<0.011). **C**, complete tumor remission is observed only with the CDDP treatment combined with STS. The arrows in (**B**) indicate the time points of individual treatments.

We extended our in vitro and in vivo observations on human mesothelioma cells to human lung adenocarcinoma cells (A549). As observed in ZL55 cells, serum starvation of A549 cells did trigger Chk2 activation, p53 accumulation and phosphorylation of histone H2AX (γ-H2AX), which is also an ATM target (Figure 4A). However, activation of ATM and AMPK was not detectable (data not shown). This might be due to different kinetics in ZL55 versus A549 cells. Indeed, in serum-starved ZL55 cells at the same time point there was no difference in phospho-H2AX levels compared to untreated control. We examined the sensitization of A549-derived xenografts to CDDP by STS in vivo as well. No significant effect on tumor growth by STS or CDDP alone was observed (Figure 4B). However, the combined treatment with CDDP and STS resulted in 65% reduction of tumor burden three weeks after treatment (Figure 4B) and complete remission of tumors without recurrence in 40% of animals treated with the combination. No complete remission occurred in any other groups at the end of the 40 weeks follow-up period (Figure 4C). Therefore, STS also sensitizes human lung adenocarcinoma xenografts to CDDP.

### In normal cells serum starvation results in proliferation arrest and protection against CDDP toxicity

**Figure 5:**
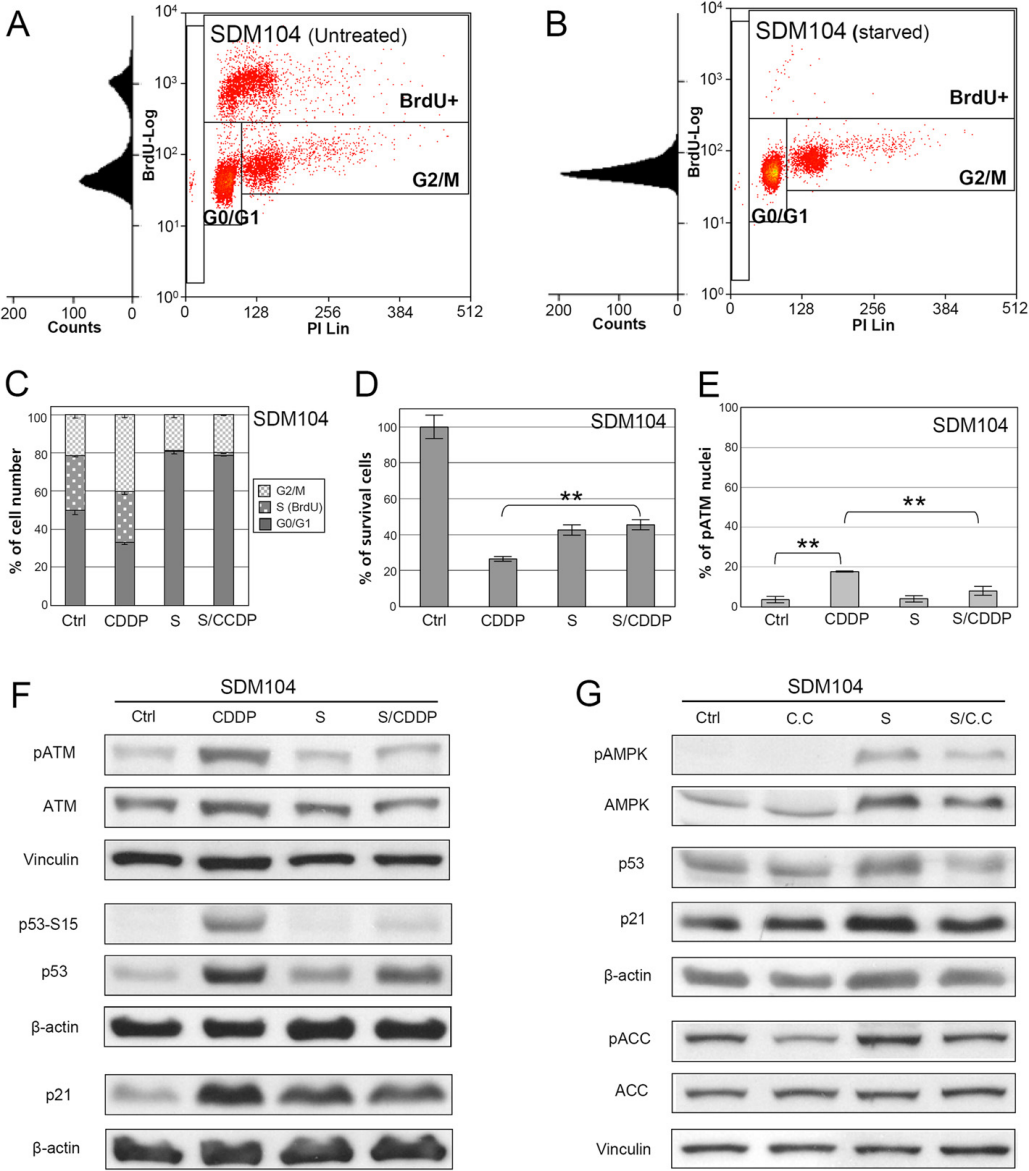
**Serum starvation induces proliferation arrest in normal SDM104 cells and protects them from CDDP cytotoxicity in vitro.** Flow cytometry analysis of SDM104 cells of untreated (**A**) or serum-starved for 24 hours (**B**). **C**, the quantification of flow cytometry results of SDM104 cells of the untreated control, or treated with CDDP alone, serum starvation alone, or both together is shown (n=3). **D**, MTT assays (n=6) were performed to examine the viability of SDM104 cells of the untreated control and those treated by CDDP alone, serum starvation alone (S), or both together in vitro (** P<0.002). **E**, the quantification of the results of anti-pATM-Ser1981 immuno-staining in SDM104 cells of untreated control, treated by CDDP alone, serum starvation alone, or both together is shown (** P<0.002, n=3). **F**, Western blot results with antibodies against pATM-Ser1981 (pATM), p53-Ser15 (p53-S15), p53 and p21 in protein extracts from the untreated control, or treated with CDDP alone, serum starvation alone, or both together are shown. **G**, Western blot results with antibodies against pAMPK, AMPK, p53, p21, pACC and ACC in protein extracts from untreated control, treated with Compound C (C.C) alone, serum starvation alone, or both together. β-Actin and vinculin were used as loading controls.

We used cultured primary human mesothelial SDM104 cells to investigate the effects of serum starvation on the response of normal cells to CDDP. In contrast to cancer cells where serum-starvation only partially reduced S-phase cells, FACS analysis of SDM104 cells showed that serum starvation alone (24 hours) resulted in complete abolishment of S-phase (BrdU-positive) cells and induction of a cell cycle arrest in G0/G1-phase, compared with untreated control (Figure 5A-C). Survival of SDM104 cells growing in the continuous presence of serum was reduced by 74% by CDDP treatment compared to untreated control cells (Figure 5D). In contrast, survival of serum starved SDM104 cells after exposure to CDDP was reduced only by 55% compared to untreated control cells, which was similar to reduction in survival observed after serum starvation alone, indicating a significant (P<0.002) increase of CDDP tolerance induced by serum starvation in normal SDM104 cells. The serum starvation-mediated protective effects from CDDP toxicity were also observed in two additional normal primary human cell cultures (Additional file 1: Figure S4).

In agreement with the ATM activation status, CDDP exposure induced activation of p53 (phosphorylation and protein accumulation) and p21 accumulation (Figure 5E and 5F). Consistent with the increased CPPD tolerance of serum-starved normal cells, CDDP-induced activation of ATM was suppressed by serum starvation (Figure 5E and 5F, and Additional file 1: Figure S5B and S5D). Although serum starvation alone did not induce activation of ATM, it triggered accumulation of p53 and the cell cycle inhibitor p21 (Figure 5F).

In order to investigate the mechanism responsible for p53 and p21 activation upon serum-starvation, we tested whether AMPK was activated by serum starvation in normal cells as well. Serum starvation induced pAMPK-Thr172 in SDM104 cells, which was confirmed by the increased phosphorylation of ACC (Figure 5G). The serum starvation-induced accumulation of p53 and the up-regulation of p21 in normal cells were inhibited by the AMPK inhibitor compound C (Figure 5G), suggesting that activation of AMPK is required for serum starvation-induced upregulation of p53 and p21. Together, these results demonstrate that serum starvation induces in normal cells the activation of AMPK thereby initiating a p53/p21-mediated proliferation arrest, which confers resistance to CDDP-induced cytotoxicity.

## Discussion

**Figure 6:**
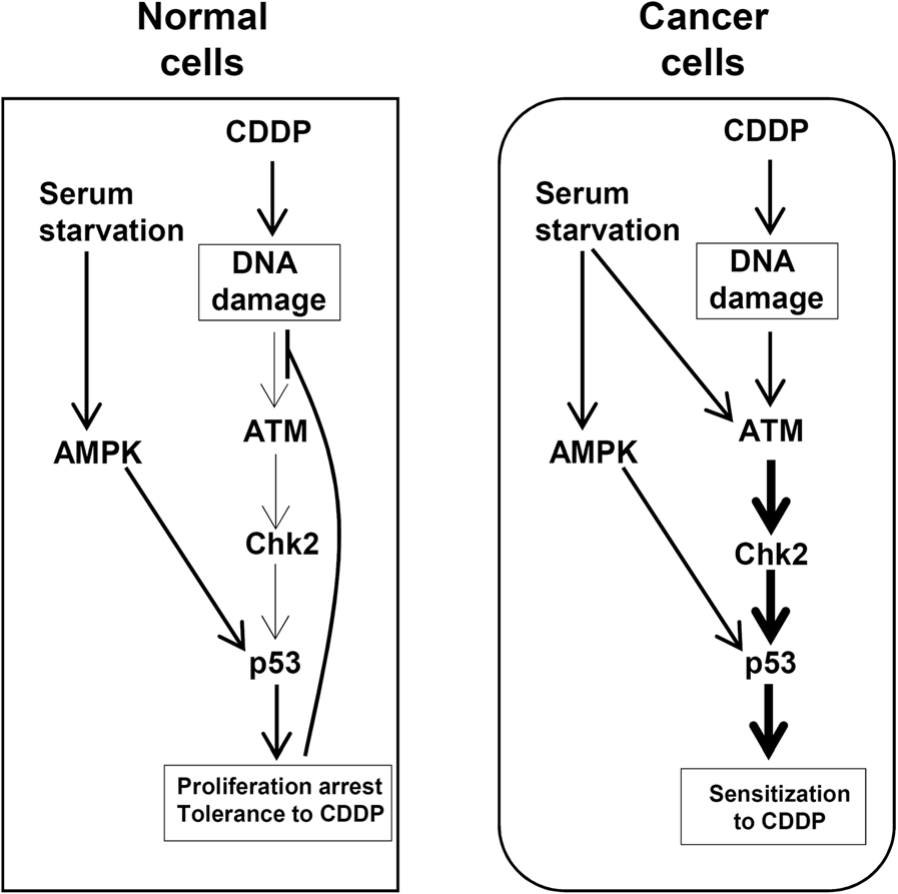
**Starvation selectively sensitizes human cancer cells to CDDP.** In normal cells, serum starvation activates AMPK, which stabilizes p53 resulting in cell proliferation arrest. The processing of CDDP-DNA-adducts in proliferating cells results in DNA damage. Proliferation arrest may contribute to the inactivation of DNA damage response thus the tolerance of normal cells to CDDP. In cancer cells, serum starvation also activates ATM/Chk2/p53 signaling. The combination of CDDP with serum starvation results in the enhanced activation of the signaling and sensitizes cancer cells to CDDP.

Our study reveals that serum starvation sensitizes cancer but not normal cells to CDDP treatment. Indeed, serum starvation activates in cancer but not normal cells ATM/Chk2/p53 signaling pathway. The latter is also activated, as expected, after CDDP treatment. Challenging serum starved cancer cells with CDDP triggers the hyper-activation of ATM/Chk2/p53 signaling resulting in sensitization of cancer cells to CDDP (Figure 6). This observation is in line with the recently developed concept that overloading stress may exhaust the cellular stress response pathways thereby sensitizing cancer cells to chemotherapy [5].

One of the potential reasons for ATM/Chk2/p53 activation by serum starvation may be due to a temporary loss of coordination between the cell proliferation driven by oncogenic mutations and the paracrine growth factors–stimulated cell growth, thus resulting in cellular stress. For example, proliferation in the absence of serum is known to lead to depletion of the nucleotide pool [25], and the latter has been associated with altered DNA replication dynamics and genomic instability which trigger the activation of DNA damage response [26]. In this context it is noteworthy that pemetrexed, an agent known to deplete nucleotide pools in cancer cells [27], improves the efficacy of CDDP therapy in the clinic [28,29].

Another possibility for ATM/Chk2/p53 activation by serum starvation is the induction of oxidative stress, which directly oxidizes ATM thereby being independent of DNA damage induction [15]. However, serum starvation did not induce the general oxidative stress marker heme oxygenase-1 [30] in both ZL55 and A549 cancer cell lines tested (Additional file 1: Figure S6), ruling out oxidative stress being involved in starvation-induced CDDP sensitization. Nevertheless, this mechanism could possibly be responsible for ATM activation in HCT116 cells where low expression levels of the ATM-activating MRN-complex protein MRE11 have been described [31,32]. Understanding the mechanism of serum starvation-induced ATM activation in cancer cells is beyond the scope of the present study and future work will address this issue.

It is noteworthy that serum starvation in cancer cells induced ATM and AMPK-mediated stabilization of p53 only, whereas CDDP treatment triggered both phosphorylation of p53 at Ser15 and stabilization of the protein. Although Ser15 phosphorylation generally stabilizes the p53 protein, Ser15-phosphorylation-unrelated p53 stabilization has also been reported and potential mechanisms include acetylation and methylation. However, an acetylation-dependent mechanism was excluded because of the absence of change of the Lys373 and Lys320 acetylation status of p53 under starvation condition (data not shown). Methylation dependent mechanisms remain to be explored.

In contrast to cancer cells, serum starvation-activated AMPK triggers in normal cells the stabilization of p53 and elevation of p21 thus resulting in a cell cycle arrest. It is known that DNA replication is blocked by CDDP-DNA-adducts, which can lead to DNA replication fork breakdown and subsequent DNA damage accumulation in replicating cells [33,34]. Therefore, the observed reduction in DNA replication upon serum starvation can explain the enhanced CDDP tolerance in normal cells (Figure 6). Consistently, DNA damage response is not activated after CDDP treatment in serum-starved normal cells.

The outcome of combined STS/CDDP treatment, i.e. tumor growth delay and/or tumor eradication, is similar to a recent observation by Lee et al., where sensitization of cancer cells to several chemotherapeutic drugs was induced by low glucose and low serum level in vitro and STS in vivo. Inhibition of IGF1 signaling was required for starvation-mediated protection of normal cells and sensitization of cancer cells to therapeutic drugs [22,23].

We show here an additional mechanism mediated by the negative regulatory signaling of AMPK, which is activated under stress conditions, e.g. nutrient starvation, to suppress proliferation [35]. We observed that serum starvation activated AMPK in both cancer and normal cells. Additionally, AMPK activity was necessary for the stabilization of p53 in response to serum starvation in both cancer and normal cells. Thus, our study reveals that in addition to the inhibition of proliferation-enhancing IGF1 signaling pathway, the activation of the AMPK signaling cascade, which suppresses proliferation, is also involved in the starvation-induced protection of normal cells and sensitization of cancer cells to CDDP treatment.

Although the design of our in vivo study does not allow extrapolating that the same mechanisms observed in vitro are operative in vivo, we nevertheless observed that STS sensitized xenografts of mesothelioma and lung cancer to CDDP treatment. Prolonged fasting is now implemented in the treatment of several diseases including rheumatic diseases and chronic pain syndromes [36], indicating beneficial effects [36]. In the context of cancer treatment, STS is well-tolerated in animal models and reduces the side effects of various chemotherapy regimens [22,24]. A case series report suggested that STS may also be applicable to cancer patients [37].

## Conclusions

In normal cells, serum starvation-activated AMPK stabilizes p53 and p21 resulting in a proliferation arrest. Serum starvation thereby protects normal cells from CDDP toxicity. In cancer cells, serum starvation additionally activates the ATM/Chk2/p53 stress response signaling pathway, which is also induced by CDDP treatment. The function of AMPK is required for maintaining this signaling. Therefore, combining CDDP treatment with serum starvation results in the hyper-activation of ATM/Chk2/p53 signaling pathway, thus sensitizing cancer cells to CDDP. In line with these in vitro observations, combining CDDP treatment with short-term food starvation in vivo delays tumor growth and induces tumor regression. Thus, our study indicates that STS may enhance the efficacy of CDDP-based chemotherapy.

## Methods

### Cell Cultures and Reagents

Human mesothelioma ZL55 [38], lung carcinoma A549 [39] cells, the colorectal carcinoma cell lines HCT116 40.16 (p53+/+) and HCT116 379.2 (p53−/−) [17], and primary normal human mesothelial SDM104 [40] cells were cultured in M199 (Invitrogen)/MCDB106 (Sigma) (1:1) mixed medium supplemented with 15% FCS, 10 ng/ml EGF, and 0.4μg/ml hydrocortisone as described [41]. The M199/MCDB106 medium without supplements was used for serum starvation assays. All cell lines used in this study were authenticated by fingerprinting (Microsynth, Balgach, Switzerland). CDDP (1667 μM saline solution, EbewePharma) was used for all in vitro and in vivo experiments. The CDDP concentration was 8 μM for all in vitro experiments unless otherwise indicated. For the in vitro treatments, cells were incubated with CDDP for 16 hours. Ku55933 and Compound C (Merck) were used at concentrations of 5 μM and 20 μM, respectively. To investigate the effects of serum starvation, CDDP treatment started 8 hours after the beginning of serum starvation and lasted 16 hours in starvation medium. Colony formation assays and MTT cell proliferation assay were performed as described [42,43]. Due to their limited growth ability, proliferation of normal cells was examined with MTT assay.

### Immunostaining and Western blot

For immunostaining, cells were fixed with 4% formaldehyde in PBS for 20 minutes in room temperature. Permeabilization was done with 100% methanol at 4°C for 4 minutes. The incubations with the first and the secondary antibodies were done at room temperature for 2 and 1 hours, respectively. Stained cells were mounted with Prolong Gold Anti-fade reagent with 4^′^,6 –diamidino-2-phenylindole (Invitrogen). Images were taken with an inverse wide-field fluorescence microscope (Leica). Anti-ATM-Ser1981 antibody (Cell Signaling) for immunostaining, anti-ATM-Ser1981 (Epitomics) for Western, anti-ATM (2C1) (GeneTex), anti-Akt, anti-phospho-Akt (Ser473), anti-phospho-AMPKα (Thr172) (40H9), anti-AMPKα, anti-Chk2, anti-phospho-p53-Ser15, anti-S6and anti-phospho-S6 (Cell Signaling), anti-phospho-Chk2-T68 (R&D System), anti-phospho-H2AX (γ-H2AX) (Millipore), anti-p53 and anti-p21 (Santa Cruz) were used according to the product instructions. Western blot analysis was performed as described [44]. For Western against ATM, protein extracts were run in 5% SDS-polyacrylamide gel while the rest were run in 13.5% SDS-polyacrylamide gel. Western blots were quantified using ImageJ.

### Flow cytometry (FACS) analysis

Before harvesting, cells were exposed to 10 μM BrdU for one hour. FACS samples were prepared as described [45]. After anti-BrdU antibody (BD Biosciences) and propidium iodide (PI) (Sigma) staining, FACS was performed with FACS Calibur (FACScan, BD Biosciences) and data was analysed with Summit v4.3software.

### In vivo experiments

All animal experiments were approved by Kantonales Veterinätamt Zürich and performed in accordance with the ethical principles and guidelines for experiments on animals of Swiss Academy of Medical Sciences. 8 week-old CD1 female nude mice (Charles River and Harlan) were randomly divided into groups. ZL55 cancer cells or A549 cancer cells in 100 μl PBS (0.5x10^6^ cells per animal) were subcutaneously injected into the left flank side of the mice. Tumor volume was measured by calliper and calculated with the formula: Width^2^×Length/2 [46]. Tumor growth in untreated animals was used as control. Treatments started when the tumor volumes reached 30±17 mm^3^. CDDP (3 mg/kg) was injected i.p. once per week for three weeks. Short-term fasting was implemented 32 hours prior and 16 hours post-injection with water ad libitum. During the treatment, animals were monitored routinely for body weight loss and general behaviour. Tumor growth and animal weight were recorded once per week for at least 16 weeks. Animals were sacrificed when any of the following signs for severe suffering was observed: the tumor volume reached 500 mm^3^; body weight loss was observed for 4 consecutive days; loss of more than 15% body weight; sign of severe sickness, for example, reduced mobility and eating, ruffled hair, and hunched back posture.

### Statistical analysis

Mann–Whitney or two-tailed *t*-Test was performed.

## Competing interests

There is no competing interest to disclose.

## Authors’ contributions

YS, EF, and TM conceived and designed the experiments. YS performed the experiments. TM assisted the flow cytometry assays and KO was involved in the initiation of the animal assay. YS, EF, TM, KO, and MP analysed the data. YS and EF provided and prepared the reagents/materials/analysis tools for the experiments. YS, EF and RS wrote the manuscript. TM and MP corrected the manuscript. All authors read and approved the final manuscript.

## Pre-publication history

The pre-publication history for this paper can be accessed here:

http://www.biomedcentral.com/1471-2407/12/571/prepub

## Supplementary Material

Additional file 1 **Figure S1. Serum starvation and CDDP both activate ATM in ZL55 cancer cells and when combined result in an enhanced activation of the ATM.** Anti-phosphoATM-Ser1981 (pATM) immuno-staining of untreated ZL55 cells (A) and those treated with 8 μM CDDP alone (B), serum starvation alone (C), or both together (D) are shown. In (A-D), images of anti-pATM staining (in red) are in left, and images of DAPI staining in middle while on the right are the overlap. “S” in (C) and (D) stands for serum starvation. **Figure S2.** p53 is knockout in HCT116^p53−/−^cells. No p53 was detected in the protein extract from CDDP-treated HCT116^p53−/−^ cells while it was induced in p53-proficient HCT116 cells. β-Actin was used as loading control. **Figure S3.** STS with water ad libitum is tolerable for animals. Animals regained most of the lost body weight during the next day after starvation. **Figure S4.** Serum starvation protects normal cells from CDDP cytotoxity. MTT assays were performed after primary normal cell cultures LP9, SDM104 and SDM85, which was established from a normal pleural tissue received from a patient undergoing cancer unrelated thoracic surgery (this study was approved by the Zurich University Hospital ethic committee and a written informed consent was obtained from the patient), were treated with CDDP alone, serum starvation alone or both together (* for P<0.002; ** for P<3.0x10^-5^). CDDP8 and CDDP20 stands for 8 μM and 20 μM CDDP, respectively. **Figure S5.** Serum starvation suppressed the CDDP-induced activation of ATM in normal cells. Anti-phosphoATM-Ser1981 (pATM) immuno-staining of untreated SDM104 cells (A) and those treated with 8 μM CDDP alone (B), serum starvation alone (C), or both together (D) are shown. In (A-D), images of anti-pATM staining (in red) are in left, and images of DAPI staining in middle while on the right are the overlap. “S” in (C) and (D) stands for serum starvation. **Figure S6.** Serum starvation does not induce the expression of oxidative stress marker, HO-1 in ZL55 and A549 cancer cells. Western blot results with antibodies against HO-1 for protein extracts from untreated control and those treated with CDDP alone, serum starvation alone, or both together are shown for ZL55 (A) and A549 (B) cells. β-Actin was used as loading control. Click here for file
