# Supplementary material for: Starvation-induced activation of ATM/Chk2/p53 signaling sensitizes cancer cells to cisplatin
Source: BMC Cancer. 2012 Dec 4;12:571. doi: 10.1186/1471-2407-12-571 (PMC3527202; doi:10.1186/1471-2407-12-571)
Supplement: Additional file 1 — Figure S1. Serum starvation and CDDP both activate ATM in ZL55 cancer cells and when combined result in an enhanced activation of the ATM. Anti-phosphoATM-Ser1981 (pATM) immuno-staining of untreated ZL55 cells (A) and those treated with 8 μM CDDP alone (B), serum starvation alone (C), or both together (D) are shown. In (A-D), images of anti-pATM staining (in red) are in left, and images of DAPI staining in middle while on the right are the overlap. “S” in (C) and (D) stands for serum starvation. Figure S2. p53 is knockout in HCT116p53−/−cells. No p53 was detected in the protein extract from CDDP-treated HCT116p53−/− cells while it was induced in p53-proficient HCT116 cells. β-Actin was used as loading control. Figure S3. STS with water ad libitum is tolerable for animals. Animals regained most of the lost body weight during the next day after starvation. Figure S4. Serum starvation protects normal cells from CDDP cytotoxity. MTT assays were performed after primary normal cell cultures LP9, SDM104 and SDM85, which was established from a normal pleural tissue received from a patient undergoing cancer unrelated thoracic surgery (this study was approved by the Zurich University Hospital ethic committee and a written informed consent was obtained from the patient), were treated with CDDP alone, serum starvation alone or both together (* for P<0.002; ** for P<3.0x10^-5^). CDDP8 and CDDP20 stands for 8 μM and 20 μM CDDP, respectively. Figure S5. Serum starvation suppressed the CDDP-induced activation of ATM in normal cells. Anti-phosphoATM-Ser1981 (pATM) immuno-staining of untreated SDM104 cells (A) and those treated with 8 μM CDDP alone (B), serum starvation alone (C), or both together (D) are shown. In (A-D), images of anti-pATM staining (in red) are in left, and images of DAPI staining in middle while on the right are the overlap. “S” in (C) and (D) stands for serum starvation. Figure S6. Serum starvation does not induce the expression of oxidative stress marker, HO-1 in [file 1471-2407-12-571-S1.pdf]

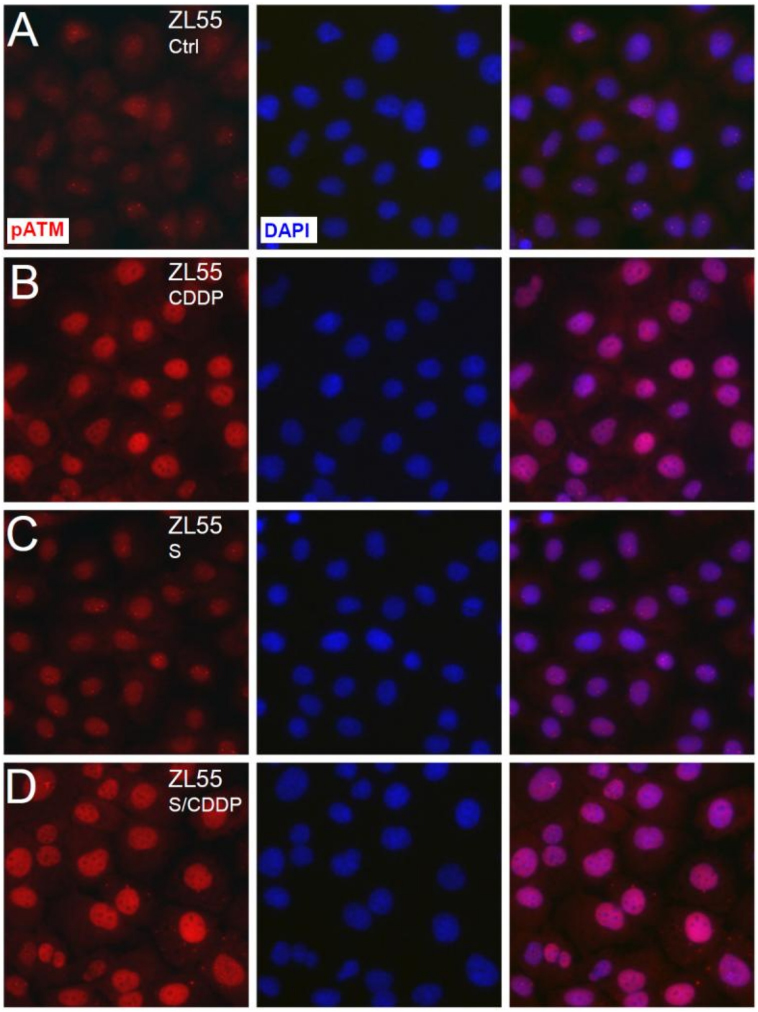

Figure S1. Serum starvation and CDDP both activate ATM in ZL55 cancer cells and when combined result in an enhanced activation of the ATM. Anti-phospho ATM-Ser1981 (pATM) immuno-staining of untreated ZL55 cells (A) and those treated with 8μM CDDP alone (B), serum starvation alone (C), or both together (D) are shown. In (A-D), images of anti-pATM staining (in red) are in left, and images of DAPI staining in middle while on the right are the overlap. “S” in (C) and (D) stands for serum starvation.

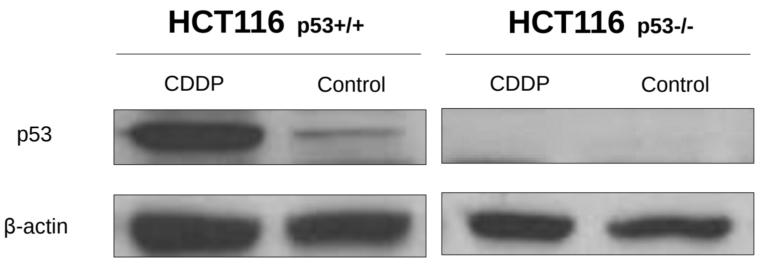

Figure S2. p53 is knockout in HCT116$^{p53-/-}$ (1) cells. No p53 was detected in the protein extract from CDDP-treated HCT116$^{p53-/-}$ cells while it was induced in p53-proficient HCT116 cells. β-Actin was used as loading control.

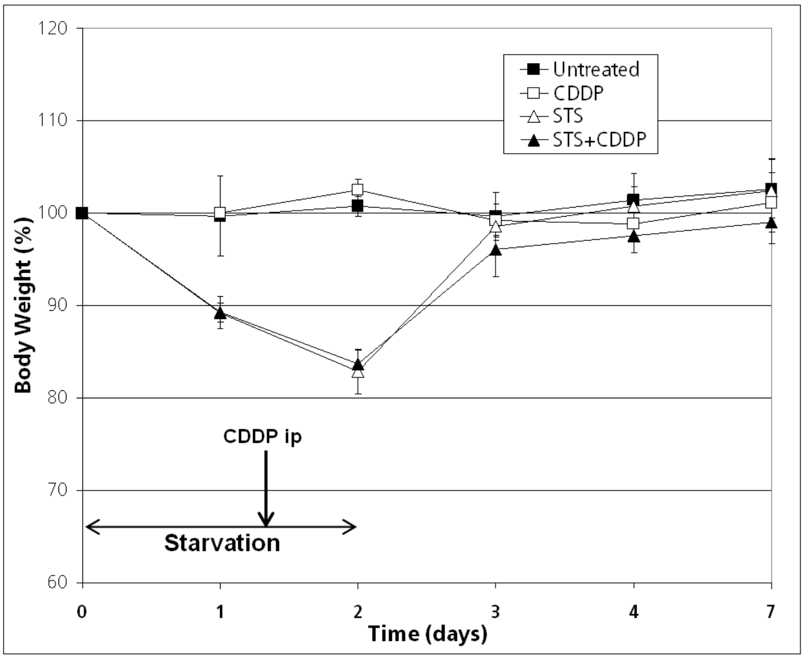

Figure S3. STS with water *ad libitum* is tolerable for animals. Animals regained most of the lost body weight during the next day after starvation.

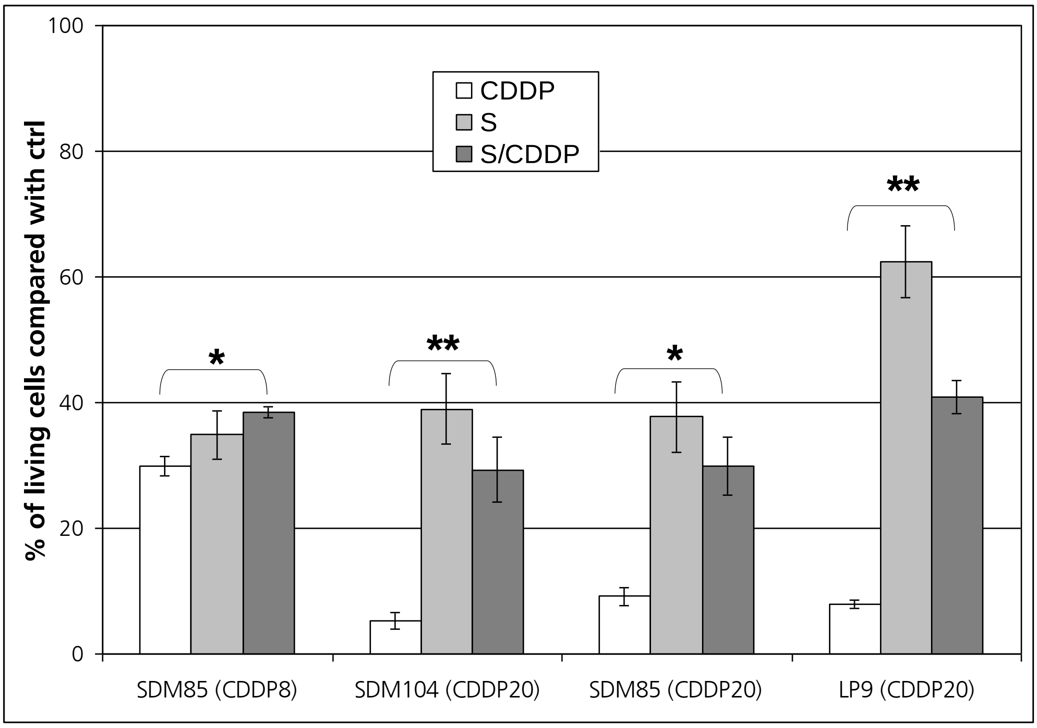

Figure S4. Serum starvation protects normal cells from CDDP cytotoxity. MTT assays were performed after primary normal cell cultures LP9 (2), SDM104 and SDM85, which was established from a normal pleural tissue received from a patient undergoing cancer-unrelated thoracic surgery (this study was approved by the Zurich University Hospital ethic committee and a written informed consent was obtained from the patient), were treated with CDDP alone, serum starvation alone or both together (* for $P<0.002$; ** for $P<3.0x10^{-5}$). CDDP8 and CDDP20 stands for 8µM and 20 µM CDDP, respectively.

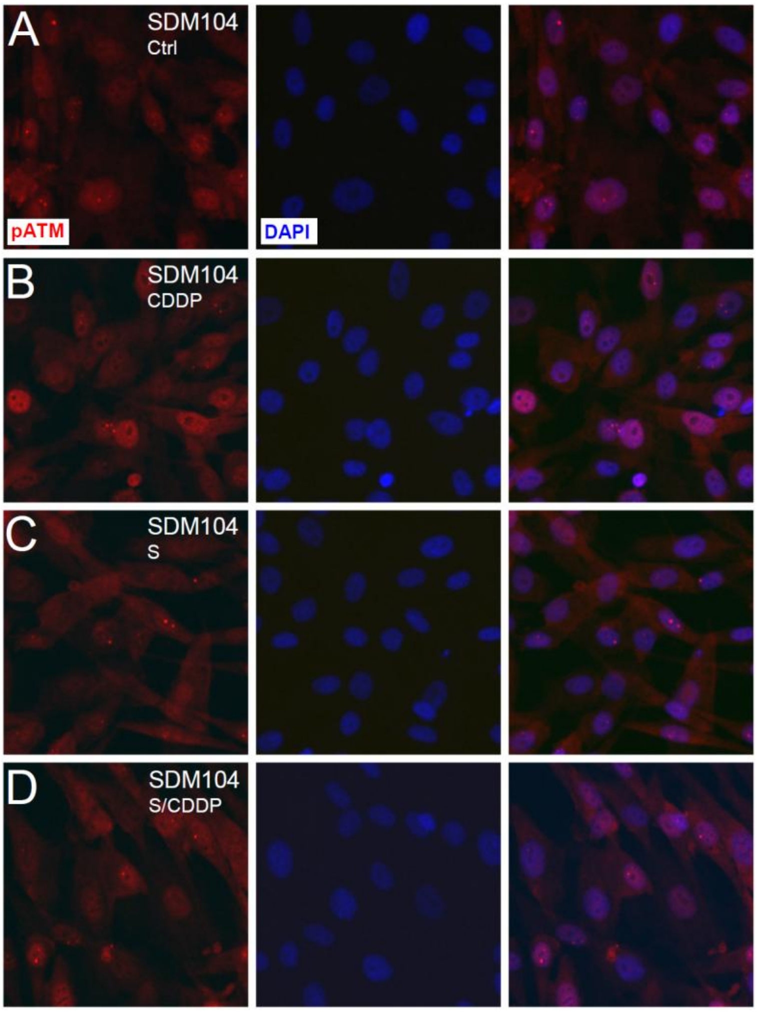

Figure S5. Serum starvation suppressed the CDDP-induced activation of ATM in normal cells. Anti-phospho ATM-Ser1981 (pATM) immuno-staining of untreated SDM104 cells (A) and those treated with 8μM CDDP alone (B), serum starvation alone (C), or both together (D) are shown. In (A-D), images of anti-pATM staining (in red) are in left, and images of DAPI staining in middle while on the right are the overlap. "S" in (C) and (D) stands for serum starvation.

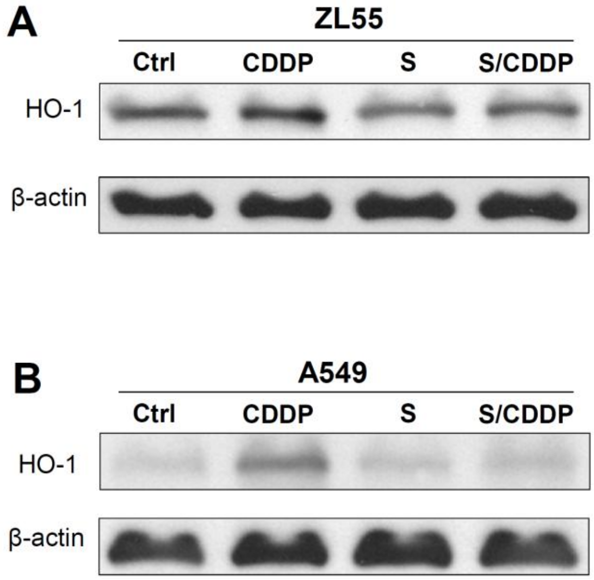

Figure S6. Serum starvation does not induce the expression of oxidative stress marker, HO-1 in ZL55 and A549 cancer cells. Western blot results with antibodies against HO-1 for protein extracts from untreated control and those treated with CDDP alone, serum starvation alone, or both together are shown for ZL55 (A) and A549 (B) cells. β-Actin was used as loading control.

Reference:

1. Bunz F, Dutriaux A, Lengauer C, Waldman T, Zhou S, Brown JP, et al. Requirement for p53 and p21 to sustain G2 arrest after DNA damage. Science. 1998;282:1497-501.
2. Dickson MA, Hahn WC, Ino Y, Ronfard V, Wu JY, Weinberg RA, et al. Human keratinocytes that express hTERT and also bypass a p16(INK4a)-enforced mechanism that limits life span become immortal yet retain normal growth and differentiation characteristics. Mol Cell Biol. 2000;20:1436-47.
